# When Should We Adopt EUS-Guided Radiofrequency Ablation in Pancreatic Neuroendocrine Tumors?

**DOI:** 10.3390/jcm12144581

**Published:** 2023-07-10

**Authors:** Matteo Marasco, Domenico Galasso, Alberto Larghi, Francesco Panzuto

**Affiliations:** 1 Digestive Diseases Unit, Sant’Andrea University Hospital, ENETS Center of Excellence, Via di Grottarossa 1035, 00189 Rome, Italy; matteo.marasco@uniroma1.it; 2 Unité de Gastro-Entérologie Service de Médicine Interne, Hôpital Riviera-Chablais, 1847 Rennaz, Switzerland; domenico.galasso@hopitalrivierachablais.ch; 3 Digestive Endoscopy Unit, Fondazione Policlinico Universitario A. Gemelli IRCCS, 00168 Rome, Italy; alberto.larghi@policlinicogemelli.it; 4 Department of Medical-Surgical Sciences and Translational Medicine, Sapienza University of Rome, Via Giorgio Nicola Papanicolau snc, 00189 Rome, Italy

Pancreatic neuroendocrine neoplasms (PanNENs) are rare and heterogeneous diseases that account for less than 2% of all cases of pancreatic cancer and only 30% of digestive neuroendocrine neoplasia, even if their incidence and prevalence continue to rise globally [1]. Pancreatic neuroendocrine neoplasms can be divided into functional (F-PanNENs) and non-functional (NF-PanNENs) neoplasms. F-PanNENs are characterized by the early onset of specific clinical manifestations that are related to the hypersecretion of specific hormones (e.g., insulinoma, gastrinoma, glucagonoma, somatostatinoma, etc.) [2]. On the other hand, NF-PanNENs, which represent the large majority of PanNENs (about 70–90%), do not express any specific hormone-related symptoms. 

The prognosis of PanNENs is affected by several factors, including the tumor size, staging and grading, which are expressed as the Ki-67 index; this is widely considered to be the strongest prognostic factor [3]. According to the 2017 WHO classification, PanNENs are divided into well-differentiated tumors (PanNETs), which may be further classified based on the proliferative activity of NET G1 (Ki67 < 3%), NET G2 (Ki67: 3–20%), and NENT G3 (Ki67 > 20%), and poorly differentiated high-grade neuroendocrine carcinomas [4].

Until now, the mainstay therapeutic approach for localized PanNENs has been surgical resection; this is the only treatment able to remove the lesion and definitively cure the patients. Unfortunately, this surgical approach is burdened with the risks of mortality and morbidity, with the most prevalent adverse events (AEs) being pancreatic fistula (45% of cases after tumor enucleation, 14% after distal pancreatectomy and pancreatoduodenectomy and 58% after central pancreatectomy), delayed gastric emptying, and hemorrhage [5].

Small, asymptomatic, incidentally detected and well-differentiated NF-PanNETs represent a clinical challenge [6]. The management of these tumors has dramatically transformed in the last decade, with an increase in the utilization of non-operative radiological surveillance as an alternative to surgery, as per the ENETS guidelines [7]. The choice between observation and resection depends on several factors, including the tumor size, the patient’s age, comorbidities, the location of the tumor, its growth in size over time and the patient’s preference. For these reasons, this choice could be considered “a grey zone of hesitation”, in which clinicians are asked to make complex decisions that should, however, always be discussed in a multidisciplinary setting before being shared with the patients. Recent evidence suggests that non-surgical active observation via annual clinical and radiological follow-up is reasonable in tumors with a size of <2 cm, as confirmed by the preliminary data of the multi-center observational ASPEN study [8]. However, living with a tumor with malignant potential may not be easy to accept, particularly in younger patients, who ought to be observed for a long time by radiological imaging studies. Thus, the development of alternative mini-invasive therapeutic approaches with a curative intent is warranted. 

EUS-guided radiofrequency ablation (EUS-RFA) has become the preferred treatment technique based on recently available scientific evidence. Due to the ability of RFA to induce the coagulative necrosis of the selected pathological tissue with minimal injury to the surrounding normal tissue, EUS-RFA has the potential to be applied as the optimal therapeutic tool, balancing high efficacy and a favorable risk profile. 

From a technical perspective, two distinct RFA devices for EUS-guided applications have been developed in order to treat PanNETs: (1) The Habib EUS-guided RFA probe (22 G needle) with a monopolar catheter can be employed in combination with commonly available radiofrequency generators, but it is, however, no longer employed in clinical practice; (2) The EUSRA EUS-RFA system (19 G needle), which is also designed with a monopolar catheter. This device is recommended for application when aiming to treat the widest area possible (through “pull-back” or “fanning” techniques of the tip), leaving a “security ring” of at least 5 mm at the periphery of the tumor to avoid thermal injuries to nearby structures [9,10,11,12].

When planning EUS-guided RFA for PanNETs, it is of paramount importance to select the patients that might benefit the most from this minimally invasive procedure compared to the standard surgical approach. Theoretically, in F-PanNETs (insulinomas), in which the goal of treatment is to cease hormonal hypersecretion syndrome, the destruction of the entire neoplastic tissue is not strictly requested; this is due to the low malignant potential of these tumors. Conversely, in NF-PanNETs, complete tumor ablation without residual neoplastic tissue is definitively required [11]. Moreover, for non-functional tumors, it is necessary to carefully stage the disease to disregard the presence of metastatic lymph-node involvement or distant metastases. 

In a systematic review and meta-analysis by Armellini et al., twenty studies (most of all retrospectives) involving a total of 183 NEN patients treated via EUS-RFA were analyzed. Overall, 196 lesions (101 functioning and 95 non-functioning) with a diameter ranging between 4.5 mm and 30 mm were included. The clinical effectiveness (disappearance of clinical symptoms for F-PanNETs and complete ablation for NF-PanNETs) was reported in 95.1% (95% CI 91.2–98.9%) and 93.4% (95% CI 88.4–98.4%) of cases, respectively [12]. 

When focusing on the specific setting of non-functioning, small (<2 cm) lesions, the expected rate of complete tumor response ranges between 70% and 100% [12].

A recent multi-center retrospective propensity matched-score study comparing EUS-RFA (89 patients) versus surgery (89 patients) for pancreatic insulinomas revealed that EUS-guided RFA was clinically effective in 95.5% of patients, without a difference compared to surgery. Notably, overall (18% vs. 61.8%) and severe (0% vs. 15.7%) AEs, as well as the hospital stay of patients (3.4 +/− 3.0 vs. 11.1 +/− 9.7 days), were significantly lower for the EUS-RFA treatment compared to surgery. Tumor relapse was observed in 16.9% of patients treated with RFA after a median follow-up of 23 months. However, a successful repeated EUS-RFA session was performed in the majority of them [13]. 

Based on these data, it is possible to conclude that EUS-RFA is a well-tolerated procedure with only a few AEs, primarily represented by non-specific mild abdominal pain, mild self-resolving acute pancreatitis, and more rarely, infection and perforation [10,14]. In order to minimize the risk of some of these events (i.e., acute pancreatitis and infection), rectal diclofenac and intravenous antibiotic administrations are usually performed prior to the procedure. Moreover, in the specific setting of insulinoma, it is strongly recommended that patients are administered a continuous 10% dextrose infusion prior to the procedure and that close glucose-level monitoring is employed, during and at least 24 hours beforehand; this is in order to prevent severe hypoglycemia, which may occur as a consequence of tumor cell necrosis [15]. Attention should be paid to patients with minute NF-PanNETs, for which the risk of progression appears negligible and should be vigilantly balanced with the risk of AEs. Indeed, in a retrospective study of 27 patients with NF-PanNETs, 4 developed acute pancreatitis that, in 3 of them, led to the formation of a collection of pancreatic fluid, which was treated with EUS-guided drainage [16]. These three patients had lesions of 10 mm, 10 mm, and 9 mm, which were likely to never evolve and thus did not require treatment.

Regarding NF-PanNETs, many oncologists and surgeons are skeptical regarding the utilization of EUS-RFA due to the impossibility of verifying the achievement of the R0 resection margins and due to uncertainty pertaining to the treatment’s long-term outcome. Only one study on 12 patients has evaluated the outcome of EUS-RFA after a mean follow up 45.6 months, and it reported only one case in which a G1 NEN reoccurred [17]. However, due to the low risk of AEs, a step-up approach that employs EUS-RFA as a first treatment modality should be considered for incidental asymptomatic NF-PanNETs with a diameter between 14–15 mm and 20 mm, and G1 or low G2 (Ki67 ≤ 5%, although cut-off is not defined); as such, surgery ought to only be undertaken in recurrent or incomplete cases [11].

In conclusion, EUS-RFA presents a novel opportunity to treat patients with small PanNETs via a minimally invasive and effective approach. The proliferating volume of available data in the setting of insulinomas supports the inclusion of this technique in the therapeutic algorithm, with its capacity to become a standard of care a real possibility when the results of an ongoing randomized controlled trial evaluating RFA are available [18]. In the clinical setting of NF-PanNENs, criteria that are applicable in the selection of patients to be treated with EUS-RFA still need to be established, despite the fact that, in our opinion, this treatment should be discussed and offered to patients. We hope that prospective data will be available as soon as the ongoing trials (RAPNEN and RFANET) are completed [19,20]. 

Nonetheless, multidisciplinary management that is able to enhance patients’ quality of care [21], as well as prompt referral to experienced centers with specific skills in RFA-related techniques, are strongly recommended before planning and implementing therapeutic strategies in these patients.
